# Interfacial Activity and Surface p*K*_a_ of Perfluoroalkyl Carboxylic Acids (PFCAs)

**DOI:** 10.1021/acs.langmuir.3c03398

**Published:** 2024-02-08

**Authors:** Ruchi Patel, Luis E. Saab, Philip J. Brahana, Kalliat T. Valsaraj, Bhuvnesh Bharti

**Affiliations:** Cain Department of Chemical Engineering, Louisiana State University, Baton Rouge, Louisiana 70803, United States

## Abstract

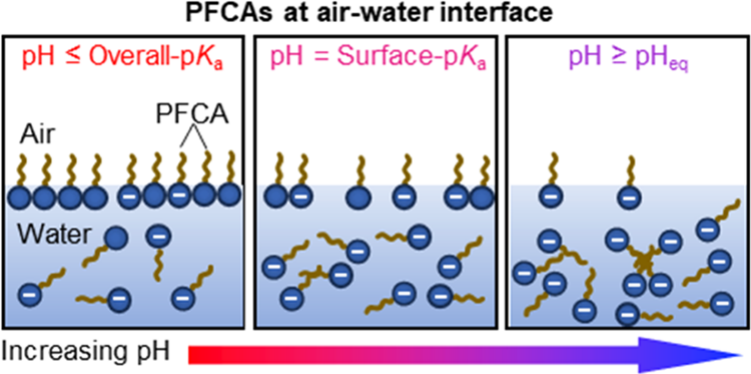

Perfluoroalkyl carboxylic acids (PFCAs) are widely used synthetic chemicals that are known for their exceptional stability and interfacial activity. Despite their industrial and environmental significance, discrepancies exist in the reported p*K*_a_ values for PFCAs, often spanning three to four units. These disparities stem from an incomplete understanding of how pH influences the ionized state of PFCA molecules in the bulk solution and at the air–water interface. Using pH titration and surface tension measurements, we show that the p*K*_a_ values of the PFCAs adsorbed at the air–water interface differ from the bulk. Below the equivalence point, the undissociated and dissociated forms of the PFCAs exist in equilibrium, driving to the spontaneous adsorption and reduced air–water surface tension. Conversely, above the equivalence point, the complete ionization of the headgroup into the carboxylate form renders PFCAs highly hydrophilic, resulting in reduced interfacial activity of the molecules. The distinction in the chemical environments at the interface and bulk results in differences in the p*K*_a_ of PFCA molecules in the bulk phase and at the air–water interface. We explore the effects of the fluoroalkyl tail length of PFCAs on their surface p*K*_a_ and interfacial activity across a broad pH range. We further demonstrate the influence of pH-dependent ionized state of PFCAs on their foamability and the rate of microdroplet evaporation, understanding of which is crucial for optimizing their industrial applications and developing effective strategies for their environmental remediation. This study underscores the potential significance of pH in directing the interfacial activity of PFCAs and prompts the inclusion of pH as a key determinant in the predictions of their fate and potential risks in the environment.

## 1 Introduction

Perfluoroalkyl carboxylic acids (PFCAs) are a class of synthetic chemicals that belong to the larger group of per- and polyfluoroalkyl substances (PFAS).^1−3^ These compounds have unique properties, including high chemical stability, interfacial activity, and resistance to heat.^4−6^ PFCAs have emerged as a matter of intense scientific concern, given their ubiquitous presence and potential environmental and human health implications.^7,8^ Due to their pronounced interfacial activity, PFCAs have been extensively employed as amphiphiles and industrial surfactants, especially in aqueous film-forming foams (AFFFs). The extensive use of PFCAs has resulted in their inadvertent deposition into the environment, having been reported in soil, groundwater, and sea spray aerosols (SSAs).^5,6,9,10^ PFCAs have been shown to alter fundamental environmental processes, such as aeration, cloud formation, and ice nucleation.^11^ The impacts of PFCAs on such environmental processes originate from their outstanding interfacial activity. Given the ubiquity of PFCAs in the environment, coupled with their ability to alter environmental processes, it becomes imperative to identify the factors that influence their interfacial activity.

Among the myriad of determinants governing interfacial phenomena, pH stands as one of the most fundamental and critical factors influencing the properties of molecules.^12−16^ Traditional investigations of surfactants have highlighted the influence of pH on surface tension, a fundamental property governed by the intermolecular interactions at the interface.^17^ For example, in amine-based gemini surfactants, the protonation of their amine groups occurs at low pH, reducing their surface activity, but they become deprotonated and more surface-active as the pH increases.^18^ Other surfactant mixtures also exhibit pH-dependent behavior, affecting their micellization and self-assembly, as exemplified in cationic fatty acid (FAs) mixed surfactant solutions, where the pH influences the oil–water interfacial tension and dilatational modulus.^14^ Similarly, the pH-dependent interfacial activity of fatty acids (FAs) has been reported extensively, where the p*K*_a_ is strongly influenced by the assembled state of the molecules.^19,20^ Note that the p*K*_a_ of a molecule is the pH at which the concentrations of the dissociated and undissociated species are equal. Interestingly, the recent work of Allen and co-workers demonstrated the differences in p*K*_a_ values of FA molecules present at the surface from the bulk.^17^ Importantly, the study established a methodology for determining surface p*K*_a_ using surface tension and pH titration measurements.

Despite the abundance of studies on conventional hydrocarbon surfactants, the pH dependence of PFCAs remains poorly understood and largely ignored. Neglecting this crucial aspect may lead to erroneous interpretations of the surface activity of PFCAs, flawed assessments of environmental fate, and inadequate risk management strategies. Moreover, the presence of significant fractions of these compounds in AFFFs^6^ and SSAs^21^ makes their pH-dependent adsorption behavior worthy of independent investigation. Given the electron-withdrawing tendency of perfluoroalkyl groups in contrast to electron-donating alkyl groups, one cannot draw direct analogies between their chemical properties and those of traditional hydrocarbon-based surfactants. We thus anticipate considerable differences in the surface activity and surface p*K*_a_ of PFCAs from their hydrocarbon counterpart, a distinction we identify in this work.

In the case of PFCAs, the pH determines the relative concentrations of their dissociated (C_*n*_F_2*n*+1_–COO^–^) and undissociated (C_*n*_F_2*n*+1_–COOH) states in water, where *n* is the number of carbon atoms in the PFCA tail. At p*K*_a_, the numbers of dissociated and undissociated PFCA molecules in the solution are equal. Hence, the lower the p*K*_a_ of PFCA, the larger the fraction of molecules present in its dissociated state at a given pH (below the equivalence point). Surprisingly, the reported p*K*_a_ values for PFCAs exhibit significant variation. For example, the p*K*_a_ of perfluorooctanoic acid (PFOA) has been reported from 0 to 4, representing nearly 4 orders of magnitude difference in hydronium ion concentration.^8,22,23^ Several reasons have been put forth to explain these wide-ranging p*K*_a_ values, including the presence of cosolvents, adsorption onto surrounding surfaces, and limitations in simulation methodologies.^23−26^ Despite numerous efforts to explain the variations in the p*K*_a_,^26^ previous studies have overlooked the role of interfacial activity of PFCAs on their p*K*_a_, an aspect that our research elucidates as a crucial factor.

In this paper, we investigate the pH dependence of the interfacial activity of PFCAs, including perfluorooctanoic acid (PFOA), and identify the impacts of pH on the ionization state of its carboxylic acid headgroup. Based on the pH titrations and corresponding surface tension measurements, we identify the overall p*K*_a_ (surface + bulk) and surface p*K*_a_ of PFCA molecules. We further determine the surface p*K*_a_ and pH-dependent interfacial activity of the homologous series of PFCAs with identical end-terminated carboxylic acid (headgroup) by increasing the number of carbon atoms in the saturated chain (tail) from *n* = 7 to 10. We demonstrate how the pH-dependent adsorption of PFOA at the air–water interface impacts the surface wetting, foamability, and evaporation rates of aqueous solutions. A holistic understanding of the pH-dependent surface tension of PFCAs will allow us to begin to devise robust and effective strategies to address their environmental persistence and mitigate potential risks to both ecosystems and human health.

## 2 Experimental Methods

### 2.1 Materials

The model PFCAs used for this study were perfluoroheptanoic acid (PFHpA—C7, purity ≥97%), perfluorooctanoic acid (PFOA—C8, purity ≥95%), perfluorononanoic acid (PFNA—C9, purity ≥97%), and perfluorodecanoic acid (PFDA—C10, purity ≥98%). These PFCAs were purchased from Sigma-Aldrich and used without further purification. Solutions of PFCAs of known concentration were prepared by dissolving them in water. Hydrochloric acid (Trace Metal grade) was purchased from Fisher Scientific, and sodium hydroxide pellets (99%) were purchased from Sigma-Aldrich. 1 M NaOH(aq) and 5 M HCl(aq) solutions were prepared and used as titrants for standard acid–base titrations. Ultrapure deionized (DI) water with a resistivity of 18.0 MΩ cm was used, and all experiments were conducted at 20 ± 1 °C with a measured relative humidity of ∼50%.

### 2.2 Methods

#### 2.2.1 Surface Tension and Contact Angle Measurements

Surface tension of PFCAs in DI water was determined using pendant drop tensiometry performed on an optical tensiometer (Biolin Scientific) and corresponding pendant droplet shape analysis. Surface tension can be determined by analyzing the contour of the droplet using the Young–Laplace equation for a liquid of known density and volume. Further details on measuring surface tension using this technique can be found in our previous publications.^27,28^ The measurements were performed on ∼10 μL of stable droplets after allowing the surface tension to equilibrate for ∼30 s. The contact angle of PFOA and water droplets on polyethylene and superhydrophobic surfaces, respectively, was measured using the sessile droplet mode of the same tensiometer.

#### 2.2.2 Surface Tension vs pH Measurements

For C7–C10 PFCAs, the surface tension vs pH data was obtained by a standard acid–base titration of a 10 mL aliquot of the PFCA solution with dropwise addition of either NaOH or HCl. The pH electrode (Hannah Instruments HI 4112) was calibrated with pH 4, 7, and 10 buffer solutions before these measurements. The surface tension was measured for an ∼10 μL solution droplet at each step of titrant addition. Effective mixing of the titrant to the solution was ensured by stirring. The measured values of surface tension are the average values reported from a set of readings recorded at 14 frames per second for about 30 s. The error bars reported for the overall p*K*_a_ are from uncertainties of pH meter measurements. The errors reported for surface p*K*_a_ are from uncertainties obtained from the surface tension data fits. This methodology is similar to that reported by Allen and co-workers for FAs.^17^

#### 2.2.3 Foamability Experiments

The aqueous solutions of PFOA and PFDA (6 mM) were prepared at different pH, and foam was produced by vigorous shaking. Foamability was quantified by calculating the ratio of the volume of foam-to-liquid i.e., *V_i_*(foam)/*V_i_*(liquid).^29^ The experiments were performed in triplicate and error bars in the data represent the standard deviation of all experimental measurements.

#### 2.2.4 Drying Droplet Experiments

For these experiments, three samples were prepared, namely, DI water (∼pH 7.0), 2 nM PFOA with pH adjusted to ∼5.6 (clean rain), and 2 nM PFOA with pH adjusted to ∼4.3 (acid rain). Four μL droplets of these samples were placed on a superhydrophobic substrate at 20 °C and ∼50% relative humidity. It was ensured that the contact angle of these droplets with the substrate was ∼150° throughout the measurement using the sessile droplet mode of the optical tensiometer. The droplets were allowed to evaporate, and droplet images were captured every 30 s using a stereoscope. Images were analyzed using the ImageJ software package to obtain the normalized size (and hence volume) of the droplet as a function of time. More details on this method and analysis can be found in our previous publications.^30,31^ The experiments were performed in triplicate, and error bars in the data represent the standard deviation of all experimental measurements.

## 3 Results and Discussion

### 3.1 Role of pH in Dictating Interfacial Adsorption of PFOA

**Figure 1:**
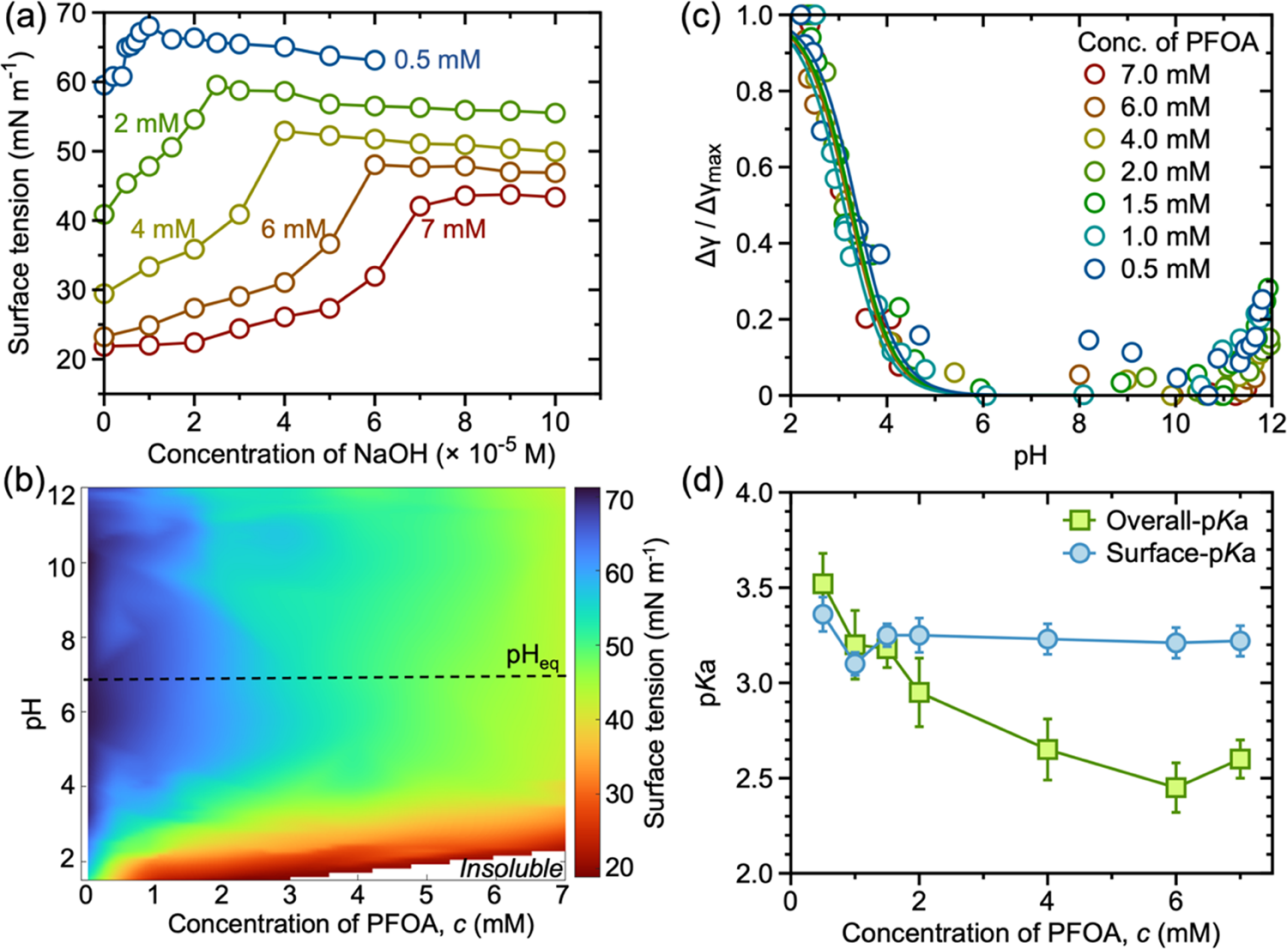
(a) Change in the surface tension of the solution with increasing amounts of added NaOH. The solutions contained increasing concentrations of PFOA as given adjacent to the respective curves. The surface tension decreases with increasing concentration of PFOA at all tested NaOH concentrations. (b) Ternary phase plot representing the codependence of surface tension on pH and concentration of PFOA. The color bar represents the experimental surface tension values for known PFOA concentrations across a wide pH range. The phase plot was generated by interpolating over one hundred seventy experimental data points. (c) Change in surface tension with pH for solutions containing increasing concentrations of PFOA. The value of change in surface tension change is normalized to the maximum surface tension change at a given concentration (see text for details). Here, the symbols represent the experimentally determined values, and lines are fits to the data using eq 1. (d) The overall and surface p*K*_a_ of PFOA solution with increasing its concentration. The two p*K*_a_ values nearly overlap at PFOA concentrations below 1.5 mM. For *c* ≥ 1.5 mM, the overall p*K*_a_ decreases with increasing concentration of PFOA, while the surface p*K*_a_ remains constant.

The amount of PFCA present in water determines the pH of the solution, and the pH governs the ionized state of the carboxylic acid headgroup and thus influences the interfacial binding affinity of the PFCA molecules. We use standard acid–base titrations to investigate the role of solution pH on the ionization of PFOA, a model PFCA. In a typical experiment, increasing amounts of 1 M NaOH (or 5 M HCl) solution were added to 10 mL of aqueous solution containing a known concentration of PFOA. After each addition, the pH and surface tension of the solution were measured using a pH electrode and an optical tensiometer, respectively, as shown in Figure 1a,b. The pH titration curves at all tested PFOA concentrations show a sigmoidal shape, characteristics of the neutralization of weak acids using a strong base, where a rapid increase in the pH is observed at the equivalence point^32^ (Figure S1). Based on the Henderson–Hasselbalch equation, the overall p*K*_a_ values were estimated using the pH at half equivalence point of the titration curve as given in Figure 1d [see Supporting Information (SI) Note S1]. The overall p*K*_a_ shows a gradual decrease with an increasing PFOA concentration (discussed later). Note that the overall p*K*_a_ measured in our experiments does not refer to the molecular p*K*_a_ of the PFCA, which is previously reported to be ∼1.0.^22,24^ The overall p*K*_a_ value obtained from the pH titrations is the p*K*_a_ value of all possible states of the PFCAs in the bulk solution, which would include aggregated states, such as micelles and discrete colloidal-sized domains. Likewise, the surface p*K*_a_ of PFCAs reported in this work is not equal to the p*K*_a_ of an isolated molecule adsorbed at the interface. While pH titrations allow for estimating the overall p*K*_a_, they do not provide any exclusive information on the p*K*_a_ of the molecules at the air–water interface.

**Figure 2:**
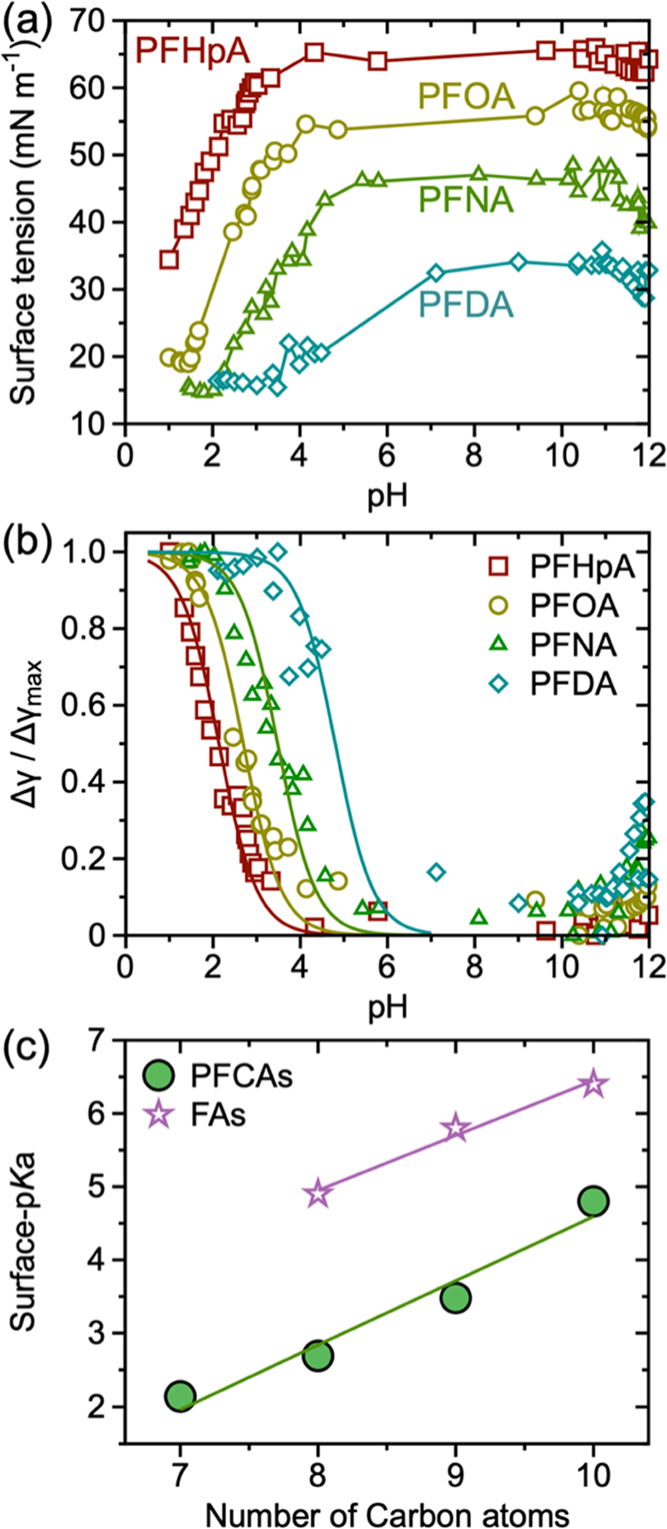
(a) Change in surface tension of PFHpA, PFOA, PFNA, and PFDA with increasing pH, demonstrating the impact of the ionized state of the headgroup on the interfacial activity of PFCAs. The concentration of all PFCAs used here was 2 mM. (b) Experimentally determined change in the surface tension with respect to the state of least interfacial activity for the homologous series of PFOA and corresponding fits (solid lines) using eq 1. The shift in the characteristic decay of Δγ/Δγ_max_ curves to the higher pH with increasing perfluoroalkyl tail length indicates an increase in the surface p*K*_a_ of the respective molecules. (c) The change in surface p*K*_a_ of PFCAs and FAs (from ref (17)), highlighting the similarity in the slope of the change in the surface p*K*_a_ with the increasing number of carbon atoms in the tail. The electron-withdrawing nature of the perfluoroalkyl tail results in a decrease in the surface p*K*_a_ of the PFCAs in comparison to the FAs for the corresponding chain length.

The undissociated form (C_*n*_F_2*n*+1_–COOH) of PFCAs is more interfacially active than its dissociated form (C_*n*_F_2*n*+1_–COO^–^). Thus, a transition of the carboxylic acid headgroup from neutral to anionic state upon increasing the pH has immediate implications for the interfacial activity of PFCAs. To quantify such an effect, we measured the air–water surface tension for PFOA solutions with their increasing concentrations and increasing pH (Figure 1a,b). In the absence of added NaOH, the surface tension of the aqueous solution decreases with an increasing concentration of PFOA. The surface tension reaches as low as 20 mN/m at 7 mM PFOA, highlighting the high interfacial activity of the molecules. The surface tension of PFOA solutions at all tested concentrations showed a gradual increase upon the addition of the base (Figure 1a). At a given concentration of PFOA, the surface tension values show only a slight decrease at pH > pH_eq_, highlighting that the ionized state of the molecules does not change significantly beyond the equivalence point. Here, pH_eq_ is the pH at the equivalence point, which is ∼7. Such observations were also made of other PFCA homologues used in the study (Figure 2a). The observed slight decrease in surface tension at pH > pH_eq_ can be attributed to the saponification of PFCAs at high pH, driving the complexation of sodium ions with the carboxylate headgroup, leading to the formation of a soap with increased interfacial activity.^33^

The codependence of surface tension on pH and PFOA concentration is shown in Figure 1b. This depiction underscores the alterations in the interfacial behavior of PFOA molecules, brought about by variations in the ratios of dissociated and undissociated acid headgroups at the air–water interface, a phenomenon intricately regulated by the pH of the solution. At a pH below the overall p*K*_a_, the dissociated and undissociated forms of PFOA remain in equilibrium, and the surfactant solution behaves as a mixture of dissociated and undissociated states showing a spontaneous adsorption at the air–water interface. This is represented in the red region of the phase plot, where the interfacial tension is very low (Figure 1b). An increase in interfacial tension is observed until pH_eq_, denoting an increase in fraction of dissociated form of the surfactant at the expense of undissociated form, leading to a gradual increase in surface tension. Above pH_eq_, the headgroup of PFOA exists solely as the carboxylate ion due to complete dissociation, which makes the molecules highly hydrophilic. This drives the desorption of the surfactant molecules from the air–water interface into the bulk of the solution (Figure 1b).

The dependence of the surface tension (γ) on pH for PFCAs allows for exclusive estimation of their surface p*K*_a_. Here, we use the methodology developed for FAs by Allen and co-workers^17^ and estimate the surface p*K*_a_ of PFOA with increasing bulk concentration (see Supporting Information, Note S2). The model assumes that at sufficiently low pH (≪overall-p*K*_a_), all molecules exist exclusively in their undissociated form, and at sufficiently large pH (≫pH_eq_), all molecules are fully dissociated.^17,34^ Under such assumptions, the change in surface tension (Δγ) of PFCA solution upon altering the pH with respect to the fully dissociated form of least interfacial activity (at pH_eq_) as the reference state, i.e., max(Δγ) is related to the surface p*K*_a_ as
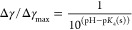
1where p*K*_a_(s) is the surface p*K*_a_ of PFCA molecules and Δγ_max_ is the maximum change in γ. The change in Δγ/Δγ_max_ with pH is shown in Figure 1c, and we use eq 1 to fit the experimental data with p*K*_a_(s) as the only free fit parameter (Supporting Information, Figure S2). Interestingly, we observe that at *c* ≤ 1.5 mM, majority of PFOA exists at the air–water interface; thus, the surface p*K*_a_ and overall p*K*_a_ are nearly identical. Upon increasing the PFOA concentration to the range *c* > 1.5 mM, the surface p*K*_a_ remains nearly constant, and the overall p*K*_a_ continues to decrease. The observed decrease in overall p*K*_a_ can be attributed to the effective increase in the number of PFCA molecules (both aggregated and individual) in the bulk solution. The formation of self-assembled structures either in bulk or at the interface (monolayers) restricts the release of H^+^ from the headgroup, thus decreasing the p*K*_a_.

### 3.2 Effect of Perfluoroalkyl Tail Length on Surface p*K*_a_

The pH dependence of the headgroup transformation and corresponding change in interfacial activity are generic behaviors across all PFCAs. We demonstrate such generality by determining the change in surface tension with pH for perfluoroheptanoic acid (PFHpA), perfluorooctanoic acid (PFOA), perfluorononanoic acid (PFNA), and perfluorodecanoic acid (PFDA). All tested PFCAs show an increase in the surface tension with increasing pH while below pH_eq_ (Figure 2a).

The qualitative similarity in the change in surface tension with pH for the homologous series of the PFCAs highlights the impact of headgroup ionization on the interfacial adsorption and activity of the molecules. Increasing the chain length of the PFCA leads to an increase in the surface p*K*_a_ (Figure 2b,c), which at first seems to contradict the recent report of Di Toro and co-workers.^24^ However, the previous work was not performed for selective determination of surface p*K*_a_. The surface p*K*_a_ is anticipated to be impacted by the chain length of the PFCA as it governs the solvation energy and aggregated/assembled state of molecules at the air–water interface, as previously shown for FAs.^17,19,35,36^ Note that the solubility of PFOA is dependent on the pH (Figure S2); however, the concentrations of PFCAs for this chain-dependent study were judiciously chosen such that the PFCA remained soluble in water for 2 < pH < 12. Regardless, the presence of nano and micronized clusters in bulk and at the interface cannot be completely ruled out, especially for PFNA and PFDA.

We compare the surface p*K*_a_ and interfacial activity of PFCAs to those of their FA counterparts. While the slope of the surface p*K*_a_ value vs the number of carbon atoms in the chain is nearly identical for the two (Figure 2c), the absolute value of surface p*K*_a_ for PFCAs is significantly lower than FAs. This difference is attributed to the electron-withdrawing nature of the fluoroalkyl tail of PFCAs over electron-donating characteristics of the hydrocarbon chain, which renders the proton of the PFCA easily released. The presence of CF_2_ groups in the tail of carboxylic acid-based molecules is known to impact the p*K*_a_ and corresponding surface properties.^37,38^ However, the distinction of PFCAs from hydrocarbon-based carboxylic acid molecules is critical in the context of environmental pollution, highlighting that the fraction of the PFCA molecules present at the interface at a given pH (<pH_eq_) is significantly higher than those of FAs.

### 3.3 Impacts on Foamability and Microdroplet Evaporation

The pH dependence of the ionized state of the PFOA headgroup has significant implications in driving their solubility in water and interfacial activity toward fluid–fluid and solid–liquid interfaces. We demonstrate such critical pH dependency by first determining the solubility limit of PFOA at various pH. We find that the solubility of PFOA in water increases with increasing pH (Supporting Information, Figure S2). Such an increase in the solubility can be attributed to the increasing hydrophilic characteristics of the carboxylate ion (dissociated form of PFCA) over the carboxylic acid functional groups (undissociated form of PFCA).

**Figure 3:**
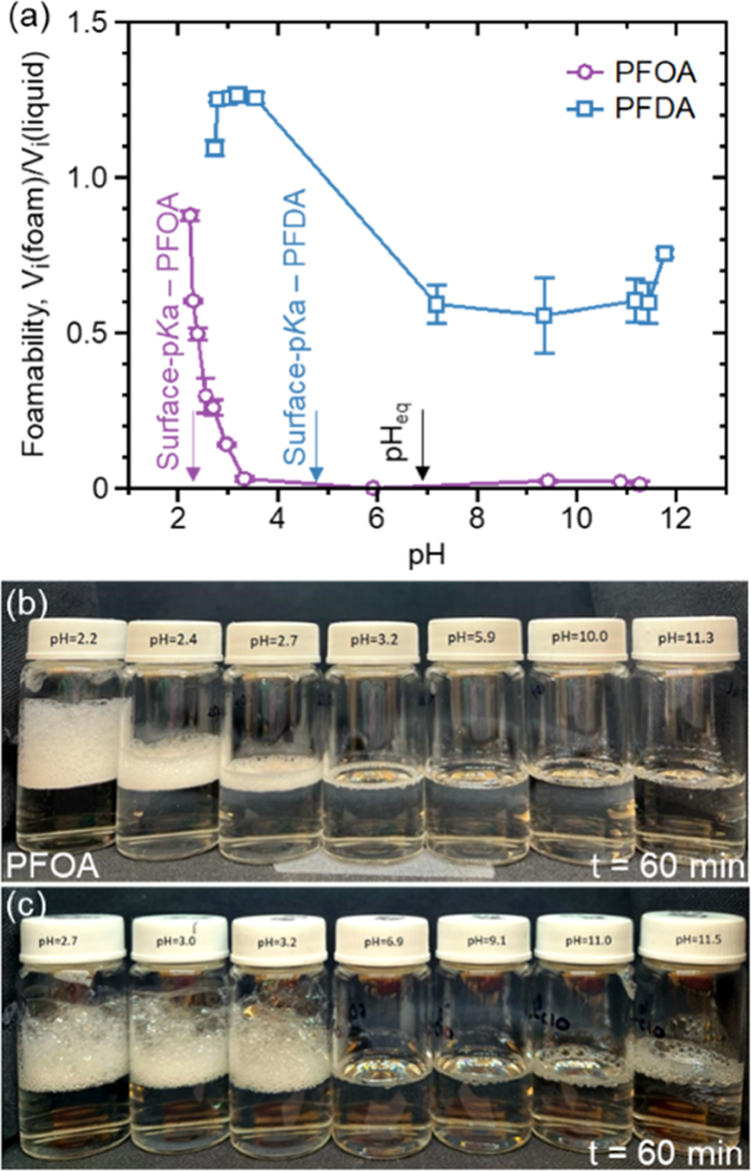
(a) Change in volume of foam formed by 6 mM aqueous solutions of PFOA and PFDA upon increasing pH. The purple and blue arrows represent the surface p*K*_a_ of PFOA and PFDA, respectively, and the black arrow represents the equivalence point of the solutions. Both solutions show maximum foamability below their respective surface p*K*_a_ due to the high interfacial activity of the undissociated headgroups. The foamability drops to a minimum value after the equivalence point, highlighting migration of molecules from the interface to the bulk of the solution. Error bars represent standard deviations from three experimental measurements. (b, c) Photographs of the change in foam volume of PFOA and PFDA solutions at various pH after 60 min. The foam volume reduces upon increasing the pH to the equivalence point due to the reduced interfacial activity of the molecules.

The reduction in the interfacial activity of PFCAs above pH_eq_ should lead to poor surface wetting ability and reduction in the foamability of the solutions. We demonstrate such behaviors using aqueous solutions of PFOA and PFDA as models. First, we measure the contact angle vs pH of a 5 μL droplet containing 6 mM PFOA on a polyethylene surface. We find that the contact angle increases with increasing pH up to pH_eq_ and decreases thereafter (Supporting Information, Figure S3). Similar pH dependency is observed for the volume of foam formed by 6 mM PFOA. Here, the foamability of a solution is represented as the ratio of volume occupied by the foam and the liquid, i.e., *V_i_*(foam)/*V_i_*(liquid). The foam volume is maximum below the surface p*K*_a_ and continuously decreases upon increasing the pH up to pH_eq_, beyond which no significant foam formation is observed (Figure 3a,b). This observation corroborates the high interfacial activity of the undissociated state of the headgroup at a low pH, followed by desorption and complete dissociation of the headgroup at pH higher than pH_eq_. The extent of reduction in foamability below pH_eq_ is significantly lesser for PFDA as compared to PFOA (Figure 3a,c). Such an observation can be attributed to the greater number of carbon atoms present in the hydrophobic perfluoroalkyl tail of the PFCA molecules, which have a relatively lower tendency to desorb from the interface. Note that the electron-withdrawing effect of the fluoroalkyl tail is usually effective only up to two carbon atoms; hence, it is the solvation energy of the tail that restricts the complete desorption of PFDA from the interface. Similar pH dependence is known for the solubility of hydrocarbon-based fatty acids in water, further confirming our conclusions on the behavior of PFCAs.^17,19^

**Figure 4:**
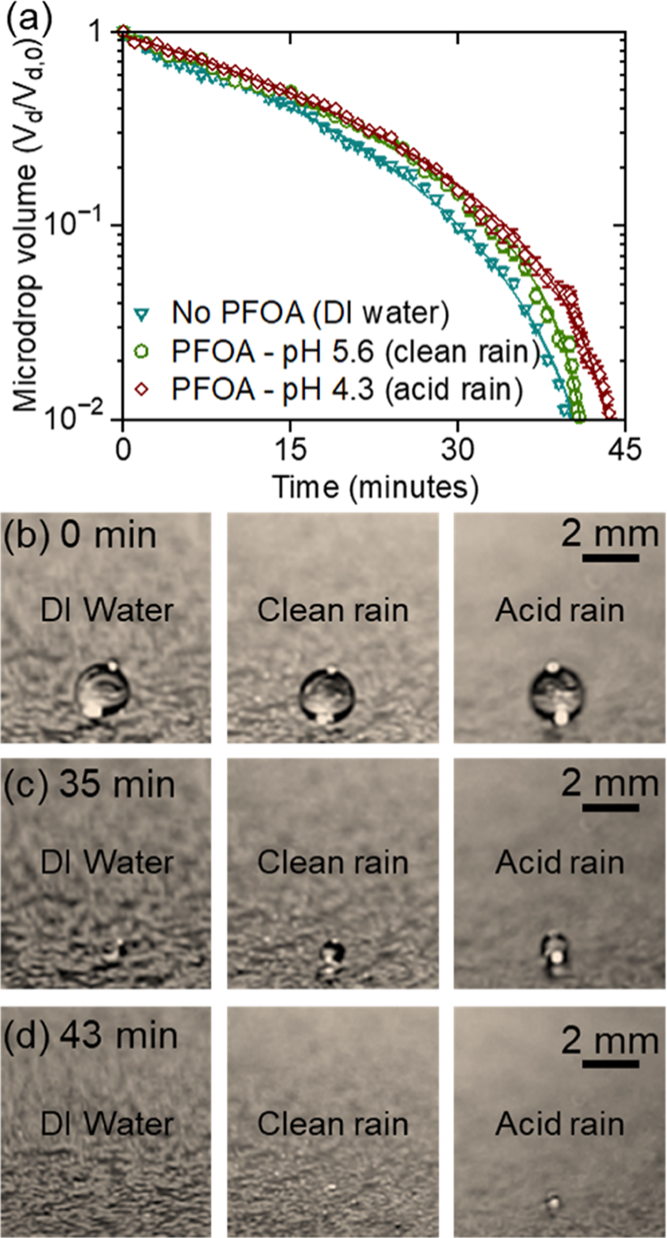
(a) Change in the volume of microdroplets with time during the evaporation process. The microdroplets were either DI water or contained 2 nM PFOA at pH 4.3 and 5.6. Here, the microdroplet volume (*V*_d_) is normalized to its respective initial volume (*V*_d,0_) at time = 0 min. The high interfacial activity of PFOA under acidic conditions suppresses the microdroplet evaporation. (b–d) Images showing the microdroplets at various times, where the microdroplet of DI water (no PFOA at pH 7.0) was the first to evaporate followed by 2 nM PFOA at pH 5.6 (clean rain) and then pH 4.3 (acid rain). The error bars represent the standard deviations of three experimental measurements.

The headgroup ionization state-dependent adsorption of PFCAs onto the air–water interface points to the potential impact of the pH on the evaporation of aqueous microdroplets. This is particularly relevant in the context of aerosols generated during the use of AFFFs as a firefighting measure, where PFCAs are one of the ingredients.^39^ This account provides a fundamental understanding of the impact of pH on the interfacial activity of PFCAs. The strong adsorption of PFCAs onto the air–water interface could influence the evaporation of the aqueous aerosols in the atmosphere. To demonstrate such an impact, we quantify the decrease in the volume of the model microdroplets containing 2 nM PFOA in water at pH 5.6 (clean rain) and 4.3 (acid rain) and compare it with droplets of DI water at pH ∼ 7.0 in the absence of PFOA. In a typical experiment, 4 μL of PFOA solution is placed on a superhydrophobic surface (water contact angle ∼150°, Supporting Information, Figure S4) and allowed to dry at 20 °C and relative humidity of ∼50% (Figure 4, and Supporting Information, Movie S1). The amount of PFOA and droplet volume was chosen based on the reported concentrations of PFOA during wet deposition in the environment at fluoropolymer plants^40^ and well-established diameter of the rain droplets (∼2 mm).^41^ The change in microdroplet size over time was measured by using a stereoscope. Initially, the decrease in volume of the microdroplets at all tested pH remains similar until ∼35 min. However, after the microdroplet volume is reduced to nearly half its initial value (after ∼80% of the total drying time), the decrease of droplet volume follows the order DI water (no PFOA at pH 7.0) > clean rain (2 nM PFOA at pH 5.6) > acid rain (2 nM PFOA at pH 4.3), as shown in Supporting Information, Movie S1. Previous reports on evaporation dynamics of surfactant-laden droplets on superhydrophobic substrates have shown differences in the drying rates after ∼90% of the total drying time.^42,43^ In our study, these minor differences in drying of droplet dynamics interestingly follow the order of interfacial activity, where PFOA at pH 4.3 has surface activity higher than that at pH 5.6 (Figure 1). The statistical analysis highlighting the differences in the drying time of the droplets is provided in Figure S5 of the SI. The adsorption of PFOA drives the formation of a dense molecular layer at the air–water interface, hindering the evaporation of water, which agrees with previous reports on water evaporation through FA and surfactant layers.^36,44^ These subtle differences observed in the evaporation dynamics corroborate the dependence of pH on the interfacial adsorption of PFOA at the air–water interface. Additionally, it highlights that under acidic atmospheric conditions, the aerosols containing PFOA (and other PFCAs) may prolong the presence of rain, fog, mist, and other atmospheric aqueous aerosol droplets.

## 4 Conclusions

In conclusion, our study of the pH-dependent interfacial behavior of PFCAs highlights the departure between the overall p*K*_a_ and surface p*K*_a_ and provides additional essential insights. The difference in the p*K*_a_ of molecules at the interface and bulk is attributed to the formation of dissimilar aggregated/assembled states and shielding of the PFCA molecules. At low pH, PFCAs spontaneously adhere to the interface, while at neutral pH, their surface activity diminishes. These findings underscore the importance of pH in influencing surface tension, solubility, and foaming stability properties. The pH of aerosolized droplets, such as sea spray aerosols, can vary considerably due to the reactions with gas-phase acidic species.^45^ Hence, our observations could have implications for understanding the chemical reactivity of PFCAs in the environment, particularly in AFFFs, SSAs, and raindrops, where the interface plays an important role. Above all, by addressing the critical knowledge gap of the pH-dependent interfacial adsorption and surface p*K*_a_ of PFCAs, we advocate for a paradigm shift. We urge researchers to incorporate this fundamental property while reporting the interfacial activity of these surfactants on their environmental presence.

## Supporting Information Available

Henderson–Hasselbach equation, model for estimation of surface p*K*_a_ for PFCAs, pH titration curve of PFOA, binary solubility-pH diagram of PFOA, contact angle vs pH of PFOA, contact angle control measurements, and statistical analysis of droplet drying experiments (PDF)
Movie of the droplet drying experiments (Movie S1) (AVI)

## Supplementary Material

la3c03398_si_001.pdf

la3c03398_si_002.avi
