# Supplementary material for: Interfacial Activity and Surface pKa of Perfluoroalkyl Carboxylic Acids (PFCAs)
Source: Langmuir. 2024 Feb 8;40(7):3651–8. doi: 10.1021/acs.langmuir.3c03398 (PMC10883055; doi:10.1021/acs.langmuir.3c03398)
Supplement: Supplementary file 1 — la3c03398_si_001.pdf [file la3c03398_si_001.pdf]

Supporting Information for

Interfacial activity and surface-p*K*a of perfluoroalkyl carboxylic acids (PFCAs)

Ruchi Patel‡, Luis E. Saab‡, Philip J. Brahana, Kalliat T. Valsaraj, and Bhuvnesh Bharti*

Cain Department of Chemical Engineering, Louisiana State University, Baton Rouge, LA 70803

*Corresponding author email: bbharti@lsu.edu

‡These authors contributed equally to the work

Supplementary Notes

Supplementary Note 1 – Henderson-Hasselbach equation for estimation of overall-pKa of PFCAs from experimental data of pH titration curve.

The overall-p*K*a of PFOA is the pH at half equivalence point as given by the Henderson-Hasselbach equation[1,2,3]. In the absence of any added NaOH, the PFOA solution attains an acidic pH due to the weak carboxylic acid headgroup, establishing the following equilibrium,

$$\mathrm{PFOA}_{(aq)} \leftrightharpoons \mathrm{PFO}^{-}_{(aq)} + \mathrm{H}^{+}_{(aq)}$$

where $\mathrm{PFO}^{-}_{(aq)}$ is the conjugate base of $\mathrm{PFOA}_{(aq)}$. In this regime $\mathrm{pH} \sim -\log\left(\sqrt{K_\mathrm{a}[\mathrm{PFOA}_{(aq)}]}\right)$, where [ ] represent the concentration, and K_a is the dissociation constant of the carboxylic acid headgroup of the PFOA. Upon the addition of base, while pH < pH_{eq} the equilibrium relation is modified as

$$\mathrm{PFOA}_{(aq)} + \mathrm{OH}^{-}_{(aq)} \leftrightharpoons \mathrm{PFO}^{-}_{(aq)} + \mathrm{H_2O}_{(l)}$$

Here the pH is given by the Henderson-Hasselbalch equation as $\mathrm{pH} = \mathrm{p}K_\mathrm{a} + \log\frac{[\mathrm{PFO}^{-}_{(aq)}]}{[\mathrm{PFOA}_{(aq)}]}$. At the equivalence point, $\mathrm{pH}_{eq} = 14 + \log\left(\sqrt{\frac{K_\mathrm{w}}{K_\mathrm{a}}[\mathrm{PFO}^{-}_{(aq)}]}\right)$, where K_w is the dissociation constant of water. Beyond the equivalence point, no buffering of the pH is anticipated, and the pH of the solution upon the addition of base is simply given as $\mathrm{pH} = 14 + \log([\mathrm{OH}^{-}_{(aq)}] - [\mathrm{PFO}^{-}_{(aq)}])$.

First, we estimate the concentration of NaOH (C_e) at the equivalence point of the titration curve as shown in Supplementary Note Fig. 1. We then calculate the pH at $C_e/2$, which we report as the overall-p*K*a, for the particular curve.

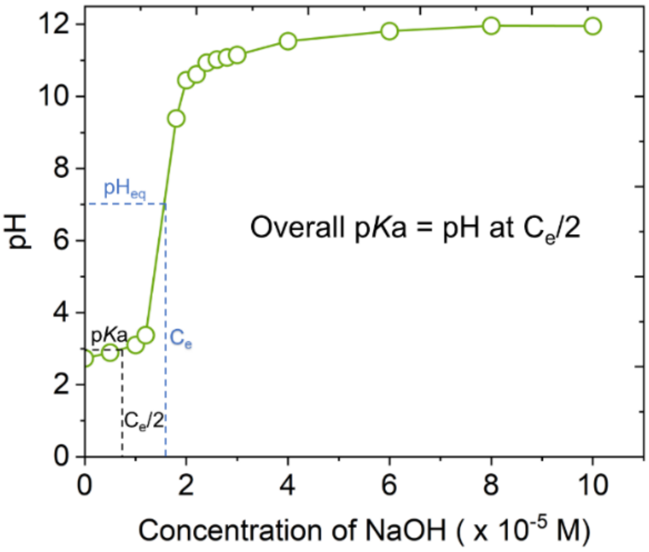

Supplementary Note Fig. 1. *pH titration curve for 2 mM PFOA. Green circles represent experimental data. Blue dashed lines indicate equivalence point and corresponding concentration of NaOH from titration. As per Handerson Hasselbach equation, the overall-pKa is the pH at half equivalence point represented by black dashed lines. The overall-pKa for this curve is ~3.*

Supplementary Note 2 – Model for estimation of surface-p*K*a using surface tension vs pH experiments.

In a recent study, Allen and co-workers presented a model relating the surface tension and surface-p*K*a for medium chained fatty acids[4]. This is modification on a model originally proposed by Cratin for the adsorption of fatty acid on oil-water interface[5]. Here we present key equations and assumptions of the model, which allows us to assess the surface-p*K*a of the PFCA molecules. At a pH below the equivalence point (at the air-water interface), following equilibrium exists:

$$\mathrm{HA} \leftrightharpoons \mathrm{A^-} + \mathrm{H^+}$$

where HA refers to the PFCA molecule. The corresponding equilibrium constant is given as

$$K_a = \frac{[\mathrm{A^-}][\mathrm{H^+}]}{[\mathrm{HA}]} \qquad (1)$$

and $\mathrm{p}K_a = -\log K_a$. The mole fraction of HA molecules is given as:

$$x_{\mathrm{HA}} = \frac{[\mathrm{A^-}]}{[\mathrm{A^-}] + [\mathrm{HA}]} \qquad (2)$$

Combining equations (1) and (2) yields

$$x_{\mathrm{HA}} = \frac{1}{1 + 10^{(\mathrm{pH} - \mathrm{p}Ka(s))}} \qquad (3)$$

Here p*K*a(s) is surface-p*K*a of the PFCA molecules at the air-water interface. If the total activity (*a*) is assumed to be the sum of dissociated and undissociated PFCAs, its value is given by

$$a = x_{\mathrm{HA}} a_{\mathrm{HA}} + (1 - x_{\mathrm{HA}}) a_{\mathrm{A^-}} \qquad (4)$$

Note that equation (4) holds true under the assumption that the PFCA species present at the interface coexist independently.

In our case, we assume the reference state of PFCA as its least interfacial active state i.e. max surface tension represented as $\max(\gamma)$. The change in surface tension of a surfactant is given by $\Delta\gamma$

$$\Delta\gamma = \max(\gamma) - \gamma \qquad (5)$$

From evaluation of plot of $\Delta\gamma$ as a function of pH (Supplementary Note Fig. 2.), we can infer that for these systems, a_{HA} = 0 in the low pH regime and $a_{\mathrm{A^-}}$ = 1 in the high pH regime, just as reported by Allen and coworkers[4].

Under such assumption[4, 6,7], the activity is given as

$$a = \frac{\Delta\gamma_{\max} - \Delta\gamma}{\Delta\gamma_{\max}} \tag{6}$$

Here $\Delta\gamma_{\max} = \Delta\gamma$ i.e. maximum in $\Delta\gamma$ with respect to the least interfacial active state which in the case of PFCA is at the pH at equivalence point (pH_{eq}). This assumption sets bounds on the numerical value of a. At low pH, molecules exhibit minimum surface activity, thus $\Delta\gamma \sim \Delta\gamma_{\max}$, rendering a ~ 0. In the high pH regime, molecules are highly surface active, thus $\Delta\gamma \sim 0$, rendering a ~ 1. Using equations 4 and 6, we can write

$$\frac{\Delta\gamma_{\max} - \Delta\gamma}{\Delta\gamma_{\max}} = x_{\mathrm{HA}} a_{\mathrm{HA}} + (1 - x_{\mathrm{HA}}) a_{\mathrm{A^-}} \tag{7}$$

Assuming at sufficiently low pH all PFCAs exist in their undissociated form HA i.e. $x_{\mathrm{HA}} = 1$ and $\Delta\gamma_{\max} = \Delta\gamma$, and at neutralization point $\Delta\gamma \sim 0$, and $x_{\mathrm{HA}} = 0$. Under these two assumptions, $a_{\mathrm{HA}} = 0$ and $a_{\mathrm{A^-}} = 1$ which can be used to rewrite equation 7 as

$$\frac{\Delta\gamma_{\max} - \Delta\gamma}{\Delta\gamma_{\max}} = (1 - x_{\mathrm{HA}}) \tag{8}$$

which simplifies to

$$\frac{\Delta\gamma}{\Delta\gamma_{\max}} = x_{\mathrm{HA}} \tag{9}$$

Using equation (3) and (9) we can get to the final relation,

$$\frac{\Delta\gamma}{\Delta\gamma_{\max}} = \frac{1}{1 + 10^{(\mathrm{pH} - \mathrm{p}Ka(s))}} \tag{10}$$

This relation assumes that lowering of surface tension with decreasing pH is the result of protonation of the PFCA (i.e. formation of HA), which has significantly higher interfacial activity (and lower surface tension) than the reference state (A^-) which is true for PFCAs. For estimating surface-p*K*a, we use equation (10) to fit our experimental data ($\Delta\gamma / \Delta\gamma_{\max}$ vs pH) with p*K*a(s) as the only free-fit parameter.

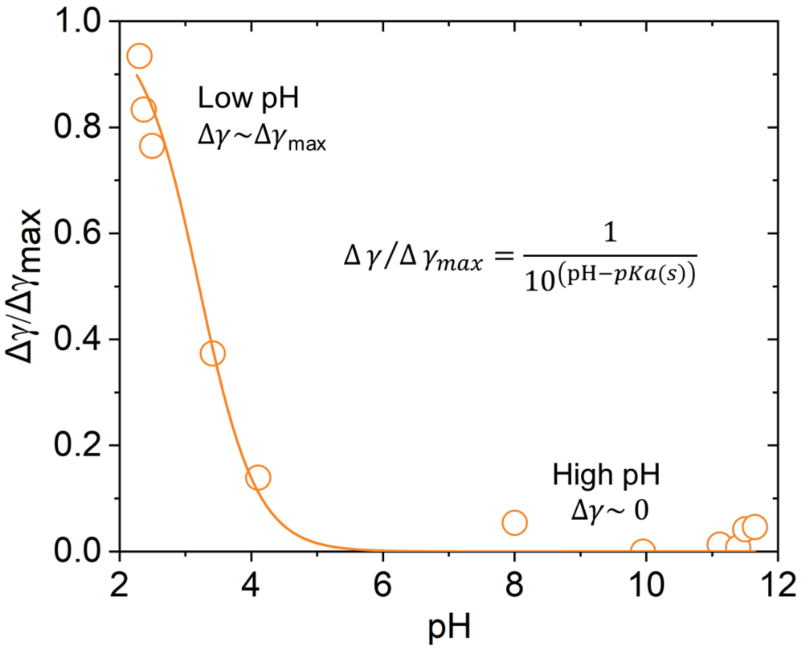

Supplementary Note Fig. 2. *Normalized change in surface tension as a function of pH for 6 mM PFOA. Orange circles represent experimental data points and the line is the fit to the data points using equation 10. The plot distinctly shows two regimes, low pH – where $\Delta\gamma = \Delta\gamma_{max}$ and high pH where $\Delta\gamma \sim 0$. The value of surface-pKa obtained by fitting the experimental data is 3.2 ± 0.1.*

Supplementary Figures

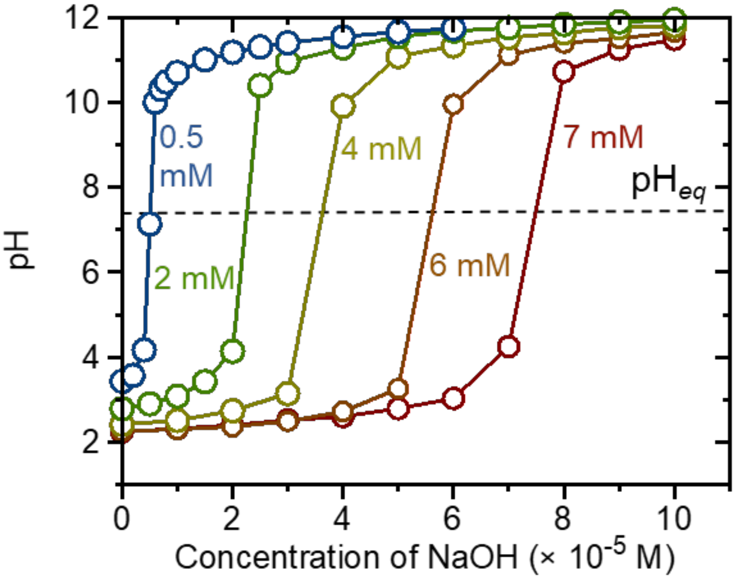

Fig. S1. *Change in pH of the solution containing increasing concentrations of PFOA with increasing added amounts of NaOH. The horizontal line represents equivalence point, pH$_{eq}$ of this pH-titration curve. The overall-pKa is estimated for each of these curves using Henderson-Hasselbalch equation, which is the pH at half equivalence point.*

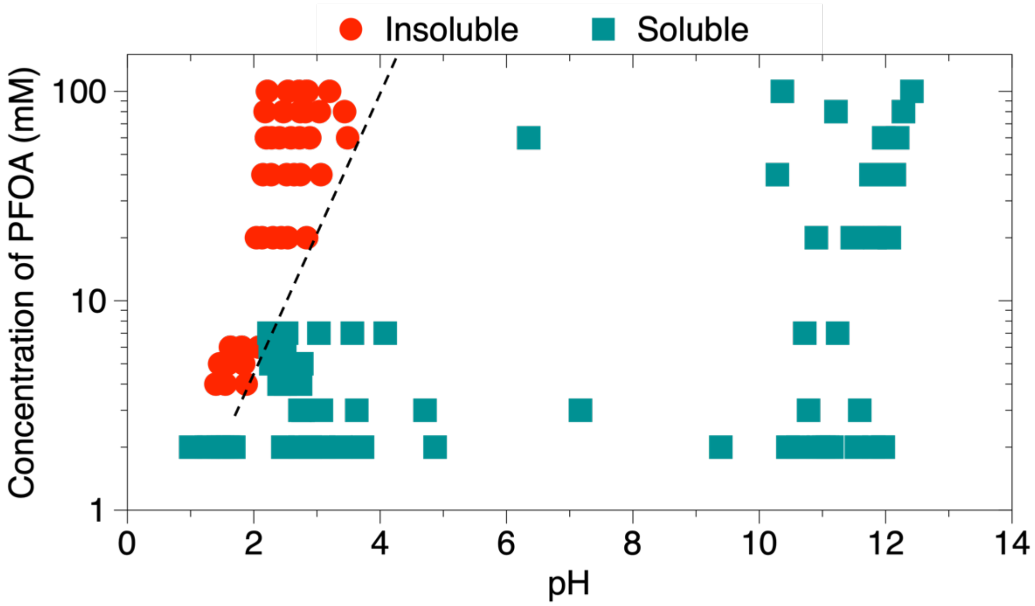

Fig. S2. *Binary diagram representing the change in solubility of PFOA in water across a wide pH range. The circles represent the insoluble state of the PFOA in water and the squares represent the soluble state.*

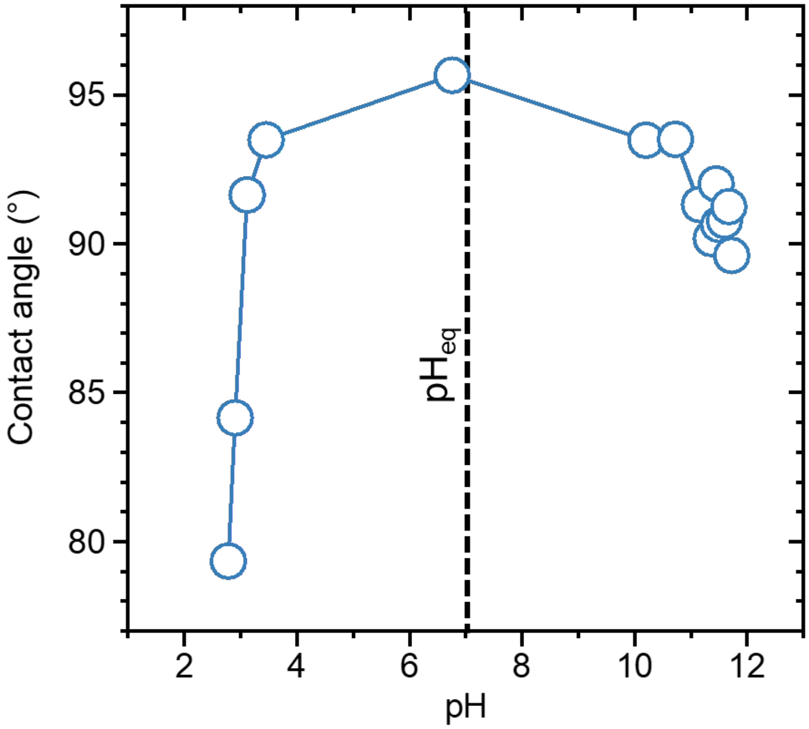

Fig. S3. *The effect of the change in pH on the contact angle of a droplet containing 6 mM PFOA onto a polyethylene surface. Increase in contact angle upto* pH_{eq} *indicates high interfacial activity of the undissociated acid headgroups, while reduction in contact angle thereafter, denotes reduction in interfacial activity due to desorption of molecules from the interface into the bulk of the solution.*

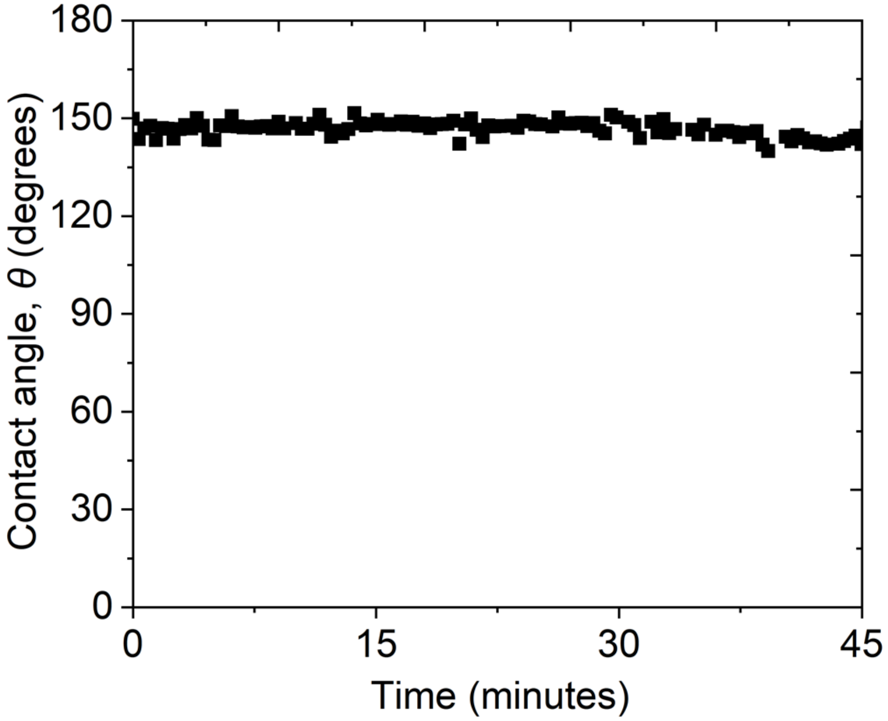

Fig. S4. *Measured contact angle, θ of a water droplet on the superhydrophobic substrate used for the droplet drying experiments. The initial droplet volume was 4 μL, and the contact angle was nearly constant throughout the drying process.*

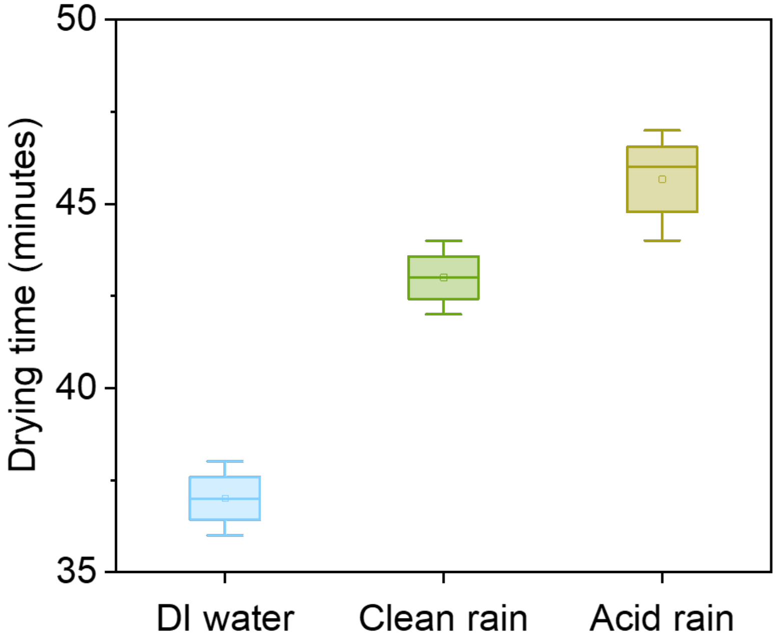

Fig. S5. *Box plot showing the droplet drying times of DI water, clean rain and acid rain. The error bars represent the standard deviation from replicate experiments. There are considerable differences in the total drying time of the three droplets at the p < 0.1 level. Post-hoc comparisons using the Tukey HSD test reveal that mean drying time for the acid rain is significantly different than clean rain (p = 0.07) and DI water (p < 0.001), and that clean rain is significantly different than DI water (p=0.002). All data sets passed Shapiro-Wilk test for normality.*

References

(1) Henderson, L. J. Concerning the relationship between the strength of acids and their capacity to preserve neutrality. *American Journal of Physiology-Legacy Content* **1908**, *21* (2), 173-179.
(2) Hasselbalch, K. The calculation of blood pH via the partition of carbon dioxide in plasma and oxygen binding of the blood as a function of plasma pH. *Biochem Z* **1916**, *78*, 112-144.
(3) Yamaguchi, A.; Namekawa, M.; Kamijo, T.; Itoh, T.; Teramae, N. Acid− base equilibria inside amine-functionalized mesoporous silica. *Analytical chemistry* **2011**, *83* (8), 2939-2946.
(4) Wellen, B. A.; Lach, E. A.; Allen, H. C. Surface p K a of octanoic, nonanoic, and decanoic fatty acids at the air–water interface: applications to atmospheric aerosol chemistry. *Physical Chemistry Chemical Physics* **2017**, *19* (39), 26551-26558.
(5) Cratin, P. D. Mathematical modeling of some pH-dependent surface and interfacial properties of stearic acid. *Journal of dispersion science and technology* **1993**, *14* (5), 559-602.
(6) Cratin, P. D. QUANTITATIVE CHARACTERIZATION OF pH-DEPENDENT SYSTEMS. *Industrial & Engineering Chemistry* **1969**, *61* (2), 35-45.
(7) Cratin, P. D. Surface and interfacial dissociation constants: apparent vs. absolute. *Colloids and Surfaces A: Physicochemical and Engineering Aspects* **1994**, *89* (2-3), 103-108.
